# Supplementary material for: Sample Preparation Method for MALDI Mass Spectrometry Imaging of Fresh-Frozen Spines
Source: Anal Chem. 2023 Oct 27;95(47):17337–46. doi: 10.1021/acs.analchem.3c03672 (PMC10688227; doi:10.1021/acs.analchem.3c03672)
Supplement: Supplementary file 1 — ac3c03672_si_001.pdf [file ac3c03672_si_001.pdf]

Supporting Information

Sample preparation method for MALDI mass spectrometry imaging of fresh-frozen spines

Kayle J. Bender[§], Yongheng Wang[‡], Chuo Ying Zhai[§], Zoe Saenz[†], Aijun Wang[#,¥], Elizabeth K. Neumann*[,§]

[§]Department of Chemistry, University of California, Davis, One Shields Avenue, Davis, CA 95616, United States

[‡]Department of Biomedical Engineering, University of California, Davis, Davis, CA 95616, United States

[†]Department of Surgery, University of California, Davis, School of Medicine, Sacramento, CA 95817, United States

[#]Center for Surgical Bioengineering, Department of Surgery, University of California, Davis, School of Medicine, Sacramento, CA 95817, United States

[¥]Institute for Pediatric Regenerative Medicine, Shriners Hospital for Children Northern California, UC Davis School of Medicine, Sacramento, CA 96817, United States

Table of Contents

Figures:

Figure S1. Mass spectra comparison for method with and without use of copper tape.
Figure S2. Labels for several tissue types of interest.
Figure S3. MALDI MSI ion images of spinal column prepared with and without cryotape.
Figure S4. Additional mass spectra for grey matter, white matter, and muscle tissue.
Figure S5. Approximate location of sampling for three spines used in Figure 6.

Tables:

Table S1. MS settings, spectrum settings, and tune settings for timsTOF fleX.
Table S2. Putative lipid assignments with *m/z* values.

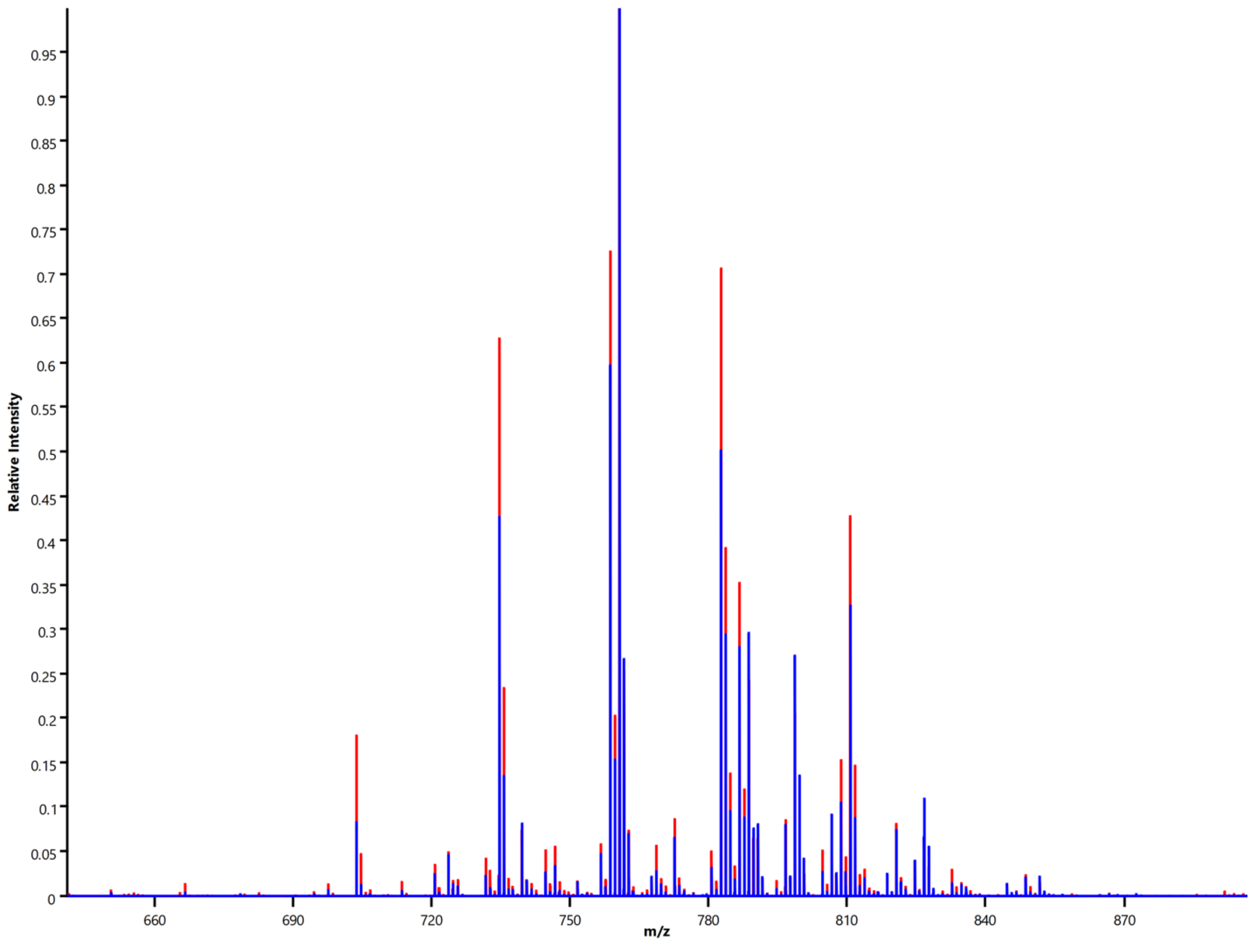

Figure S1. Mass spectra comparison for this method, with copper tape (red) and without copper tape (blue).

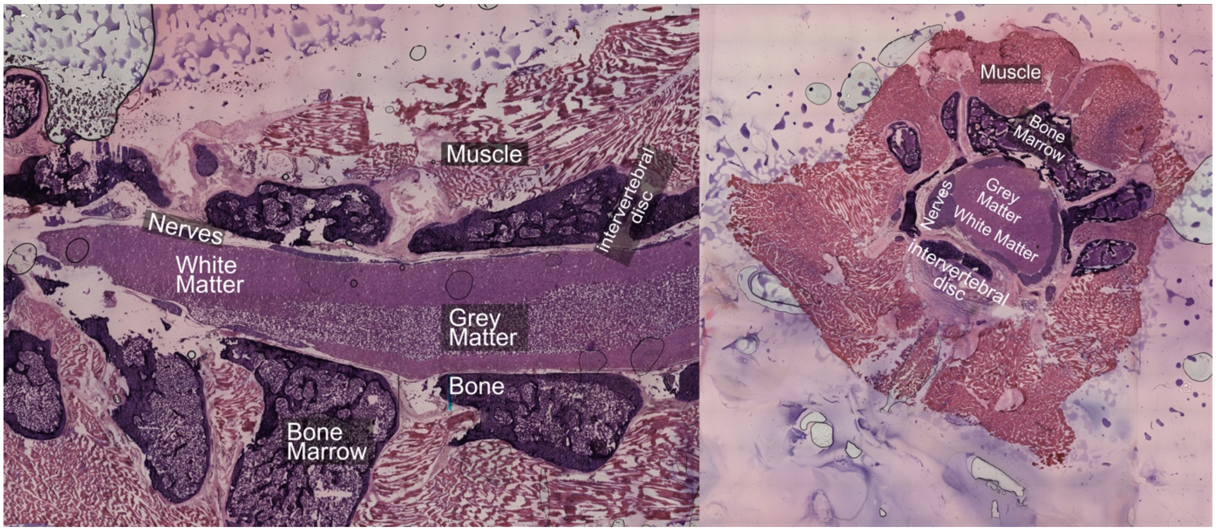

Figure S2. H&E stained sagittal and transverse sections with labels for several tissue types of interest.

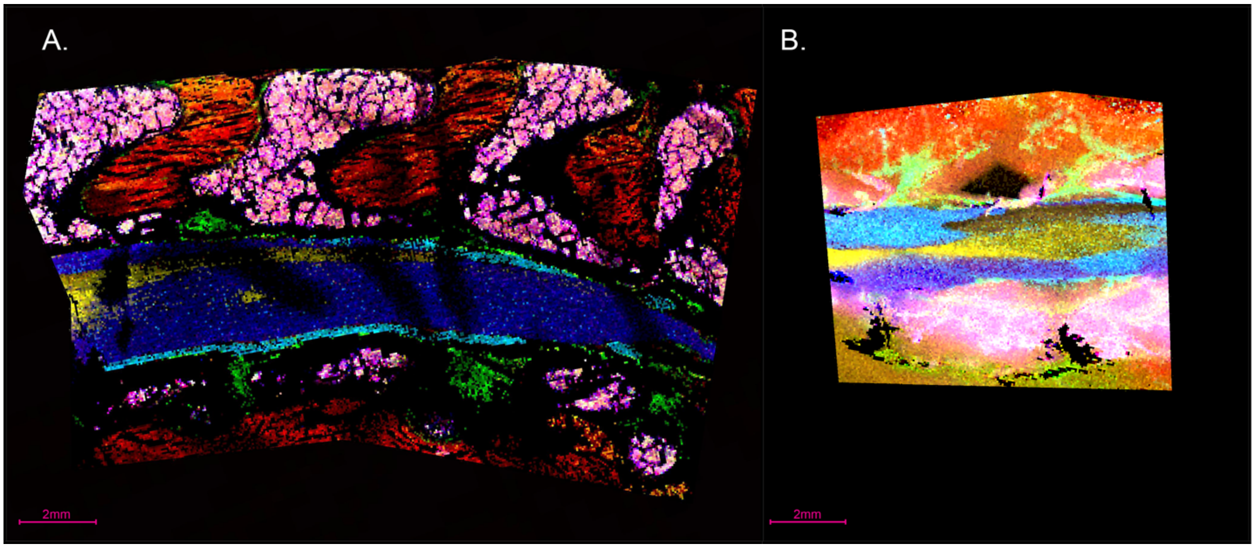

Figure S3. MALDI MSI ion images of sagittal cut fresh-frozen, undecalcified spinal column prepared using cryotape (A) and without using cryotape (B).

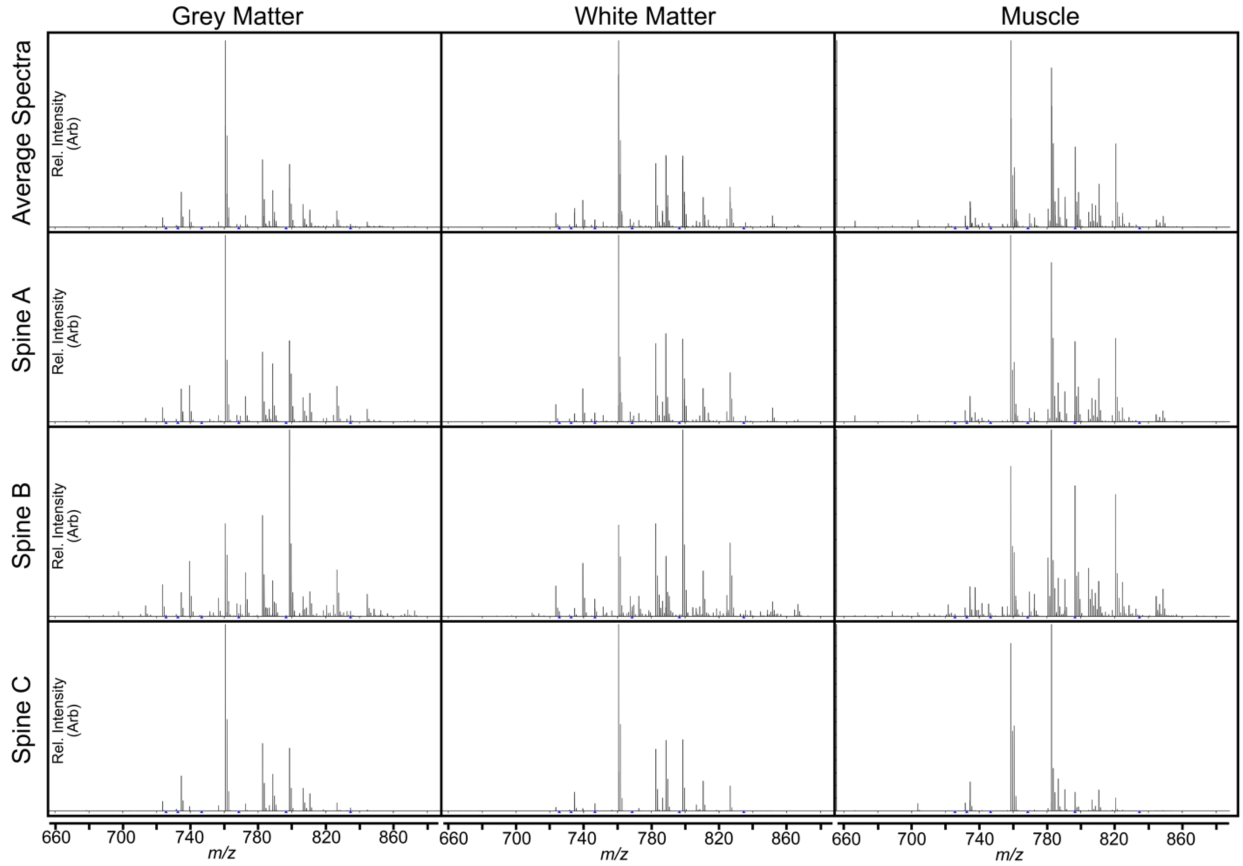

Figure S4. Mass spectra for grey matter, white matter, and muscle tissue in each of three spines and average mass spectra for spines A, B, and C (Figure 6) combined for each tissue type.

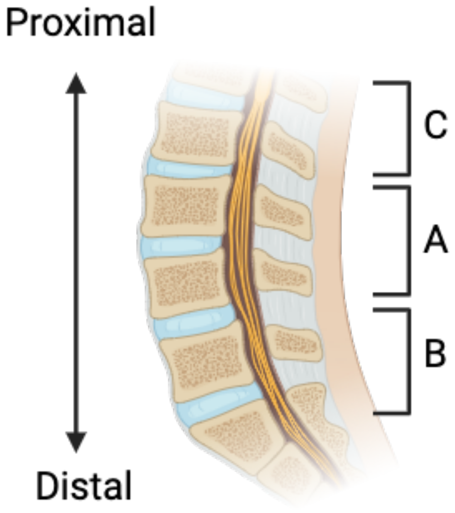

Figure S5. Approximate location of sampling for three spines used in Figure 6.

MS Settings	Scan Begin	50 *m/z*
	Scan End	1200 *m/z*
	Ion Polarity	Positive
	Scan Mode	MS
Spectrum Settings	Rate Mode	Summation
	Rate Value	165
	MS Averaging	1
Laser Settings	Laser Power	88%
	Laser Bursts	1
	Laser shots per burst	150
	Laser frequency	10000 Hz
Transfer Settings	MALDI Plate Offset	30.0 V
	Deflection 1 Delta	110.0 V
	Funnel 1 RF	400.0 Vpp
	isCID Energy	0.0 eV
	Funnel 2 RF	400.0 Vpp
	Multipole RF	400.0 Vpp
Collision Cell Settings	Collision Energy	5.0 eV
	Collision RF	500.0 Vpp
Quadrupole Settings	Ion Energy	5.0 eV
	Low Mass	500 *m/z*
Focus Pre TOF Settings	Transfer time	80.0 µs
	Pre Pulse Settings	8.0 µs
Detection Settings	High Sensitivity Detection	No
	Focus Mode	No

Table S1. MS settings, spectrum settings, and tune settings for timsTOF fleX.

Lipid Assignment	*m/z value – Observed*	*m/z value – Calculated*	Error (ppm)
**[PS(28:1)+H]⁺**	678.4361	678.4341	2.9480
**[SM(34:1;O2)+H]⁺**	703.5749	703.5748	0.1421
**[PC(30:0)+H]⁺**	706.5384	706.5381	0.4246
**[HexCer(32:1;O2)+K]⁺**	710.4919	710.4968	-6.8966
**[PA(34:1)+K]⁺**	713.4501	713.4518	-2.3828
**[PC(O-32:0)+H]⁺**	720.5897	720.5902	-0.6939
**[PA(38:5)+H]⁺**	723.4939	723.4959	-2.7644
**[SM(34:1;O2)+Na]⁺**	725.5557	725.5568	-1.5161
**[SM(36:1;O2)+H]⁺**	731.6058	731.6061	-0.4101
**[PC(32:1)+H]⁺**	732.5535	732.5538	-0.4095
**[PC(32:0)+H]⁺**	734.5695	734.5694	0.1361
**[PA(36:3)+K]⁺**	737.4516	737.4518	-0.2712
**[PA(36:2)+K]⁺**	739.4673	739.4675	-0.2705
**[SM(34:1;O2)+K]⁺**	741.5305	741.5307	-0.2697
**[PC(O-34:2)+H]⁺**	744.5901	744.5902	-0.1343
**[PC(O-34:1)+H]⁺**	746.6041	746.6058	-2.2770
**[PA(38:2)+Na]⁺**	751.5228	751.5248	-2.6613
**[PC(O-32:2)+K]⁺**	754.5187	754.5147	5.3014
**[PC(32:0)+Na]⁺**	756.5514	756.5514	0.0000
**[PC(34:2)+H]⁺**	758.5676	758.5694	-2.3729
**[PC(34:1)+H]⁺**	760.5859	760.5851	1.0518
**[PA(38:2)+K]⁺**	767.5011	767.4988	2.9967
**[PC(O-34:1)+Na]⁺**	768.5857	768.5878	-2.7323
**[SM(36:1;O2)+K]⁺**	769.5609	769.5620	-1.4294
**[PC(32:0)+K]⁺**	772.5248	772.5253	-0.6472
**[PC(34:2)+Na]⁺**	780.5513	780.5514	-0.1281
**[PC(34:1)+Na]⁺**	782.5680	782.5670	1.2778
**[PC(36:3)+H]⁺**	784.5773	784.5851	-9.9416
**[PC(36:2)+H]⁺**	786.5999	786.6007	-1.0170
**[PC(36:1)+H]⁺**	788.6163	788.6164	-0.1268
**[PE(O-38:5)+K]⁺**	790.5136	790.5147	-1.3915
**[PC(O-36:2)+Na]⁺**	794.6024	794.6034	-1.2585
**[PC(34:2)+K]⁺**	796.5243	796.5253	-1.2555
**[PC(34:1)+K]⁺**	798.5427	798.5410	2.1289
**[PC(36:4)+Na]⁺**	804.5500	804.5514	-1.7401
**[PC(36:3)+Na]⁺**	806.5675	806.5670	0.6199
**[PC(36:2)+Na]⁺**	808.5815	808.5827	-1.4841
**[PC(36:1)+Na]⁺**	810.5983	810.5983	0.0000

**[SM(42:2;O2)+H]$^+$**	813.6830	813.6844	-1.7206
**[SM(42:1;O2)+H]$^+$**	815.6968	815.7000	-3.9230
**[PE(44:12)+H-H2O]$^+$**	818.5190	818.5119	8.6743
**[PC(36:4)+K]$^+$**	820.5250	820.5253	-0.3656
**[PC(36:2)+K]$^+$**	824.5566	824.5566	0.0000
**[PC(36:1)+K]$^+$**	826.5733	826.5723	1.2098
**[PC(40:9)+H]$^+$**	828.5570	828.5538	3.8622
**[PC(38:4)+Na]$^+$**	832.5810	832.5827	-2.0418
**[PC(38:3)+Na]$^+$**	834.5967	834.5983	-1.9171
**[SM(42:2;O2)+Na]$^+$**	835.6666	835.6663	0.3590
**[SM(42:1;O2)+Na]$^+$**	837.6794	837.6820	-3.1038
**[PC(38:6)+K]$^+$**	844.5260	844.5253	0.8289
**[PC(38:5)+K]$^+$**	846.5391	846.5410	-2.2444
**[PC(38:4)+K]$^+$**	848.5565	848.5566	-0.1178
**[SM(42:2;O2)+K]$^+$**	851.6475	851.6403	8.4543
**[SM(42:1;O2)+K]$^+$**	853.6529	853.6559	-3.5143

Table S2. Lipid assignment with *m/z* values, calibrated quadratically. Putative identifications from LipidMaps database. Calculated using molecular formula of lipid[89].
